# Charlson Comorbidity Index as a Predictor of Difficult Cholecystectomy in Patients With Acute Cholecystitis

**DOI:** 10.7759/cureus.31807

**Published:** 2022-11-22

**Authors:** Ahmed A Alburakan, Sulaiman Abdullah Alshammari, Wadha Saud AlOtaibi, Jawharah Hamad Almalki, Mishary M Shalhoub, Thamer A Nouh

**Affiliations:** 1 Trauma and Acute Care Surgery Unit, Department of Surgery, College of Medicine, King Saud University, Riyadh, SAU; 2 Division of General Surgery, Department of Surgery, College of Medicine, King Saud University, Riyadh, SAU; 3 Department of Radiology, King Faisal Hospital and Research Center, Riyadh, SAU

**Keywords:** acute care surgery and trauma, complicated acute cholecystitis, cholecystectomy, acute cholecystitis, charlson comorbidity index

## Abstract

Background

The Charlson Comorbidity Index (CCI) has been validated as a predictor of overall survival and post-surgical mortality. CCI is adopted by Tokyo Guidelines as one of the main criteria in the management of acute cholecystitis. Our study evaluates the role of CCI in predicting difficult cholecystectomy.

Methods

All patients who underwent cholecystectomy for acute cholecystitis between January 2017 and September 2019 were included. CCI, Emergency Surgery Score (ESS), and American Society of Anesthesiologists (ASA) score were calculated and analyzed to assess their predictive value for difficult cholecystectomy.

Results

A total of 96 patients were included and allocated to difficult and non-difficult cholecystectomy groups. CCI was found to be a significant predictor of difficult cholecystectomy (OR 1.59; 59% CI, 1.04. 2.42; p= 0.031). Similarly, ESS was found to be a predictor tool of difficult cholecystectomy (OR 1.42; 59% CI, 1.05. 1.93; p= 0.024). There was no significant difference in adverse outcomes between the two groups.

Conclusion

CCI was able to predict a difficult cholecystectomy in our study population. However further studies are required to evaluate if it can be used as a predictor of adverse outcomes in the context of acute cholecystitis.

## Introduction

Acute cholecystitis is a common complication of gallstone disease. It could present as a serious condition that can cause life-threatening complications. Higher morbidity and mortality were reported in severe acute cholecystitis, particularly when the diagnosis is delayed [1,2]. Furthermore, the overall complications tend to increase in the presence of other comorbidities [3].

Cholecystectomy remains the definitive treatment for patients with acute cholecystitis. Cholecystectomy is one of the most performed general surgery procedures [4,5]. Nowadays, the majority of cholecystectomies are performed laparoscopically. It has been proven to be safe with fewer complications, less pain, shorter hospital length of stay, and overall better postoperative outcomes compared to the open approach [6,7]. This remains true even in the setting of acute cholecystitis [6,7]. However, the risk of intraoperative and postoperative complications still exists with potentially devastating morbidity and late consequences [8]. Multiple studies were conducted aiming to identify risk factors of difficult and complicated cholecystectomy [9-12]. Preoperative factors, such as male gender, old age, history of acute cholecystitis, and ultrasound (US) findings of wall thickening and impacted stone at the neck have been reported as predictors of difficult cholecystectomy. Long operative time, bile or stone spillage, and bile duct injury were labeled as intraoperative factors [9-12].

Anticipating difficult cholecystectomy in the setting of acute cholecystitis is important to enable the surgical team to take appropriate measures to avoid excess morbidity. Tokyo guidelines were established as a tool to diagnose acute cholecystitis and grade its severity. The guidelines have since then gained popularity and were revised to accommodate newer evidence in the management of acute cholecystitis. In the latest revision of the guidelines, which was published in 2018 (TG18), acute cholecystitis was classified into three grades based on severity [13,14]. Furthermore, the Charlson Comorbidity Index (CCI) is a method for classifying comorbidities that might alter the risk of mortality. It was used to risk-stratify patients in the flowchart for the management of acute cholecystitis [14]. CCI has been shown to be an independent predictor of surgical mortality as well as overall survival [15]. However, literature evaluating its utility in acute cholecystitis remains scarce. 

In this study, we aim to evaluate the role of CCI in predicting difficult cholecystectomy. We hypothesize that CCI can be a useful predictor in these cases.

## Materials and methods

**Figure 1:**
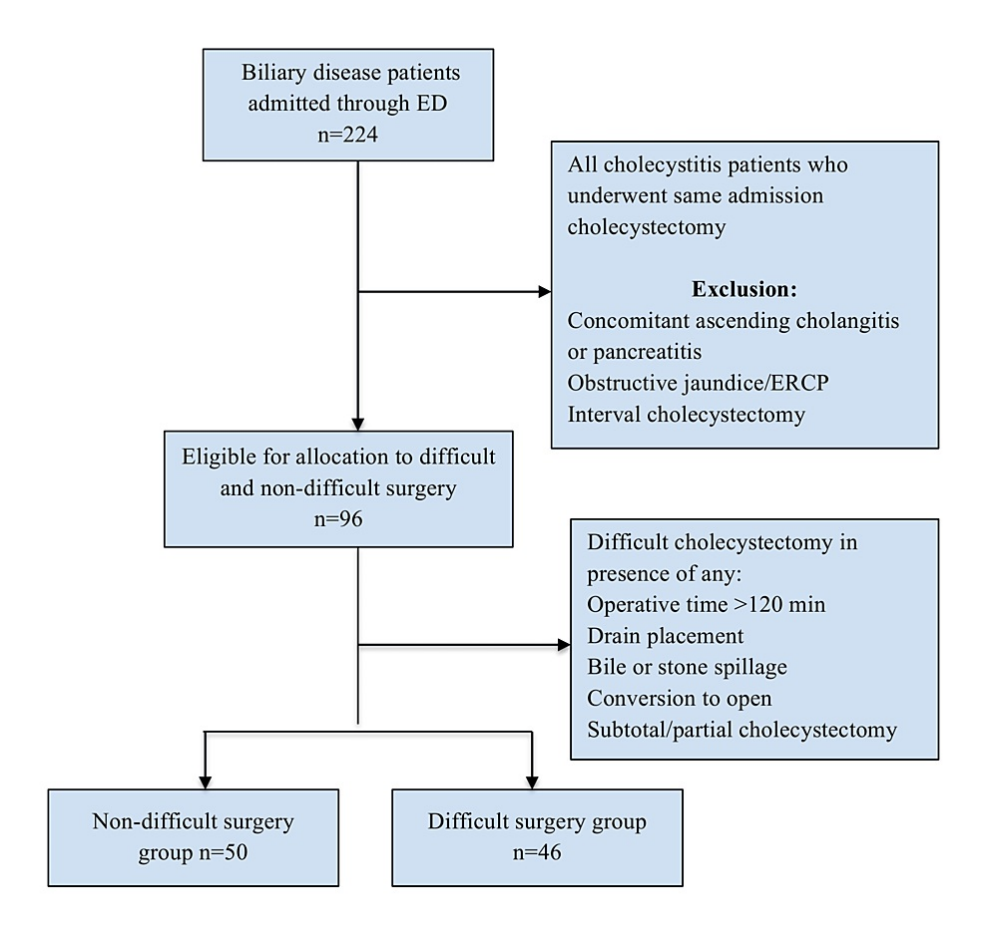
Study Flow Chart ED, Emergency Department; ERCP, Endoscopic Retrograde Cholangiopancreatography

This is a retrospective study conducted at King Saud University Medical City (KSUMC). The acute care surgery model was adopted and applied at KSUMC for more than 10 years. The acute care surgery team is dedicated and responsible for all general surgery emergencies. All patients with a biliary disease who were admitted through the emergency department between January 2017 and September 2019 were screened. All acute cholecystitis patients who underwent cholecystectomy during their admission were included. Acute cholecystitis was defined as patients presenting with a clinical picture of a white blood cell count of more than 11 or positive Murphy's sign in the presence of ultrasonic findings of thickened gallbladder wall or pericholecystic fluid. Patients with a concomitant diagnosis of pancreatitis, obstructive jaundice, and ascending cholangitis were excluded. As well as patients who underwent endoscopic retrograde cholangiopancreatography or interval cholecystectomy. Exclusion aimed to eliminate confounding factors in the assessment of our primary objective. Patients were divided into difficult and non-difficult cholecystectomy groups as shown in Figure 1.

Difficult cholecystectomy was defined based on the presence of any of the following factors [9,16]: operative time > 120 min, drain placement, bile or stone spillage, conversion to open surgery, or subtotal/partial cholecystectomy. 

At the time of presentation, baseline demographic data, vital signs, and clinical, laboratory, and ultrasound findings were collected. Operative details and postoperative complications, considering Clavien-Dindo Classification, were included to classify surgical complexity and assess outcomes, respectively. The Charlson Comorbidity Index (CCI) [15], Emergency Surgery Score (ESS) [17], and American Society of Anesthesiologists (ASA) score [18] were calculated and analyzed to assess their predictive value for difficult cholecystectomy.

Statistical analysis

Analysis was performed using STATA 14 for Windows (Stata Statistical Software: Release 14. College Station, TX: StataCorp LP). STATA is a general-purpose statistical software package developed by StataCorp for data manipulation, visualization, statistics, and automated reporting [19]. Continuous variables were presented as medians and interquartile ranges (IQRs). Variables among the two groups were compared and tested for significance using Fischer’s exact test or Mann-Whitney Wilcoxon test where appropriate. The predictive ability of CCI, ESS, and ASA scores for difficult cholecystectomy was evaluated by logistic regression.

## Results

Between January 2017 and September 2019, a total of 224 patients were admitted through the emergency department with complications of gallstone disease. Of those, 96 patients were diagnosed with acute cholecystitis and underwent cholecystectomy during their admission. A total of 128 patients fulfilled the exclusion criteria and were excluded from the analysis (Figure 1). Criteria for difficult cholecystectomy were recorded in 46 patients (Table 1).

**Table 1:** Criteria of difficult cholecystectomy

Variable	Number	Percentage
Operative time >120 min	36	37.5
Drain placement	27	28.13
Bile or stone spillage	16	16.67
Conversion to open surgery	1	1.04
Subtotal/partial cholecystectomy	2	2.08

Baseline demographics for both groups are shown in Table 2. Male sex and age ≥60 years were more prevalent in the difficult surgery group compared to the non-difficult surgery group (p-value=0.027 and p-value=0.013, respectively).

**Table 2:** Baseline demographics and clinical characteristics Data are expressed as numbers (percentages) except *median (interquartile range (IQR)). p-values <0.05 were considered statistically significant. BMI, Body Mass Index; BP, Blood Pressure; WBC, White Blood Cell; AST, Aspartate Aminotransferase; ALP, Alkaline Phosphatase; ED, Emergency Department

	Non-difficult group (n=50)	Difficult group (n=46)	p-value
Baseline demographics			
Male sex	15 (30.00)	24 (52.17)	0.027
Age > 60 years	1 (2.00)	8 (17.39)	0.013
BMI >35 kg/m2	16 (32.00)	19 (41.30)	0.344
Diabetes mellitus	5 (10.00)	6 (13.04)	0.64
Hypertension	4 (8.00)	10 (21.74)	0.082
Surgery in the past year	5 (10.00)	4 (8.70)	0.826
Vital signs on presentation			
Temperature >37.5	0 (0.00)	5 (10.90)	0.022
Systolic BP <110	14 (28.00)	7 (15.22)	0.127
Respiratory rate > 22	5 (10.00)	5 (10.90)	0.889
Heart rate >100	3 (6.00)	14 (30.43)	0.003
Shock Index > 0.9	2 (4.00)	6 (13.04)	0.147
Laboratory findings on presentation			
WBC <4.5 or >15	9 (18.00)	13 (28.26)	0.232
Hemoglobin <10	2 (4.00)	0 (0.00)	0.496
Platelets <150	0 (0.00)	3 (6.52)	0.106
Albumin <30	3 (6.00)	8 (17.39)	0.111
AST >40	24 (48.00)	18 (39.13)	0.284
ALP >125	22 (44.00)	14 (30.43)	0.143
Clinical features			
Symptom duration	3 (1-5)*	3 (1-5)*	0.73
Previous ED visit	20 (40.00)	14 (30.43)	0.327
Previous episode	44 (88.00)	30 (65.22)	0.007
Abdominal scar	24 (48.00)	11 (23.91)	0.013
Positive Murphy's sign	23 (46.00)	27 (58.70)	0.213
Gallbladder ultrasound findings			
Wall thickening > 3 mm	22 (44.00)	30 (65.22)	0.036
Pericholecystic fluid	16 (32.00)	25 (54.35)	0.026
Impacted stone	6 (12.00)	16 (34.78)	0.007

Clinical findings, lab values, and imaging

The difficult cholecystectomy group patients were less likely to have a history of previous biliary symptoms (65.2% vs 88% p-value=0.007) or abdominal scars (23.9 vs 48%, p-value=0.013). Patients in the difficult cholecystectomy group were significantly more likely to have a fever with a temperature of more than 37.5º (p-values 0.022) and tachycardia with a heart rate of more than 100 beats per minute (p-values 0.003). Ultrasound findings including gall bladder wall thickening > 3 mm (p-value=0.036), pericholecystic fluid (p-value=0.026), and impacted neck stone (p-value=0.007), were significantly more prevalent in the difficult cholecystectomy group. Clinical characteristics are shown in Table 2.

Outcomes

The overall outcomes of the two patient groups are summarized in Table 3.

**Table 3:** Overall surgical outcomes

	Non-difficult group (n=50)	Difficult group (n=46)	p-value
30 days readmission	0 (0.00)	2 (4.35)	0.227
Length of hospital stay	4 (3-6)	4 (3-7)	0.6583
Any postoperative complication	3 (6.00)	4 (8.70)	0.707

Both groups had a similar median hospital length of stay of four days. More postoperative complications were encountered in the difficult cholecystectomy group (n=4 (8.7%)) compared to the non-difficult cholecystectomy group (n=3 (6%)). However, this difference was not statistically significant (p-value=0.7). Biliary complications were seen in two cases, and both were in the difficult group. Two out of four patients were classified as Clavian-Dindo grade III in the difficult cholecystectomy group compared to none in the non-difficult group.

Two patients were readmitted within 30 days of discharge, and both were in the difficult cholecystectomy group. However, this was not statistically significant (p-value 0.227). One patient was admitted with a biliary leak while the other patient was admitted with obstructive jaundice due to a common bile duct stone. Both patients required intervention.

CCI as a predictor of difficult cholecystectomy

Using logistic regression analysis (Table 4), CCI was found to be a significant predictor of difficult cholecystectomy (OR 1.59; 59% CI, 1.04 -2.42; p= 0.031). Similarly, ESS was found to be a statistically significant predictor of difficult cholecystectomy (OR 1.42; 59% CI, 1.05 - 1.93; p= 0.024). On the other hand, ASA was found to be of no statistical significance (OR 1.33; 59% CI, 0.69 - 2.56; p= 0.379).

**Table 4:** Univariate model logistic regression analysis of the value of CCI, ESS, and ASA score as predictors of difficult cholecystectomy in acute cholecystitis p-values <0.05 were considered statistically significant. OR, Odds Ratio; CI, Confidence Interval; CCI, Charlson Comorbidity Index; ESS, Emergency Surgery Score; ASA, American Society of Anesthesiologists

	OR	95% CI	p-value
CCI	1.59	1.04-2.42	0.031
ESS	1.42	1.05-1.93	0.024
ASA	1.33	0.69-2.56	0.379

## Discussion

Acute cholecystitis remains a common presentation in emergency rooms worldwide. In our institution, admissions for complicated biliary disease constitute about 25% of all admissions [20]. With the increasing adoption of acute care surgery models in hospitals, the management of acute cholecystitis is rapidly becoming a major portion of acute care surgeons’ practice. Acute cholecystitis varies in presentation. It can range from an early inflammation of the gallbladder to severe cholecystitis with complex surgery and a complicated postoperative course [2,21]. Factors such as acute inflammation, fibrosis, and distorted anatomy were documented in the literature as predictors for difficult cholecystectomy in acute cholecystitis. Moreover, the same factors were also associated with post-cholecystectomy complications [9-11,22]. Duca et al. found that 94% of biliary duct injuries were associated with acute cholecystitis. In the same report, 67% of conversions to open surgery were due to acute cholecystitis [22]. Therefore, it is of paramount importance for the acute care surgeon to have valid tools in his arsenal to be able to predict those patients who have a high risk of complications in the setting of acute cholecystitis. Identifying high-risk patients early will enable the surgeon to better counsel these patients regarding possible interventions.

Tokyo guidelines have gained wide popularity in risk-stratifying patients with acute cholecystitis. In the TG18, a flowchart for the management of acute cholecystitis was presented to guide the treatment of acute cholecystitis and to predict the risk of complications [13,14]. In this flowchart, CCI is used to risk-stratify patients who present with acute cholecystitis. A CCI score >6 in severe acute cholecystitis indicates a high risk of complications after surgery and therefore gallbladder drainage procedures should be considered as an alternative therapeutic option [14,23]. In a cohort study, mortality was reported in 21 out of 5459 patients with acute cholecystitis, and CCI was more than 6 in 57% of those mortalities [24].

In our study, CCI proved to be an independent predictor of difficult cholecystectomy (OR 1.59; 59% CI, 1.04. 2.42; p= 0.031). This is in concordance with the scarce existing data evaluating CCI in acute cholecystitis. Notably, Bonaventura et al. have shown that age-adjusted >5 was associated with more mortality and morbidity [25]. The emergency surgery score (ESS) has been shown to predict outcomes in the setting of emergency general surgery [17,20]. ESS was found to be a valid tool in predicting difficult cholecystectomy in our patient population as well. The same cannot be said about the ASA score, which did not achieve statistical significance as an independent predictor of difficult cholecystectomy.

Interestingly, our result showed no significant difference between the two groups in overall surgical outcomes including re-admission rate, hospital stay, and postoperative complication. However, two of the complications in the difficult group were classified as Clavien-Dindo grade III in comparison to none in the non-difficult group. Moreover, the two biliary duct complications encountered in our study were in the difficult group. However, these findings were not statistically significant. Nonetheless, biliary complications are often devastating to the patients and the surgeon and carry an important clinical significance [26].

Cremer et al. assessed the effect of differences in surgical strategy comparing outcome data of two large-volume hospitals. And they reported that in the case of a difficult cholecystectomy, the surgical strategy seems to have an important impact on postoperative complication outcome [27]. Using valid tools to predict a difficult cholecystectomy can guide the surgeon to utilize all the available resources and expertise in an optimal time frame to improve the outcome of those patients. In this study, we have shown that the CCI score can be used as a predictor of difficult cholecystectomy.

It is natural to associate a difficult surgical procedure with adverse events. Difficult procedures challenge the surgical team and require the surgeon to be prepared and cautious [28]. Furthermore, with the rapid adoption of acute care surgery models and the limited access to operative time, the acute care surgeon may encounter the problem of difficult cholecystectomy at an unfavorable time. Therefore, having the tools to predict difficult procedures is very useful. We have shown that the CCI score can be used as a risk assessment tool when dealing with patients admitted with acute cholecystitis. CCI will provide valuable information that can help the surgeon identify possible difficult cholecystectomy and therefore plan ahead for these procedures to be done in a more controlled setting and with intensive monitoring in the perioperative period.

This study has some limitations such as the small sample size, the retrospective nature, and single-center enrollment. We believe further studies are required to validate tools that predict outcomes in the management of acute cholecystitis. Nonetheless, CCI has shown that it can predict difficult cholecystectomy procedures in our single-center patient cohort. It should be considered when evaluating patients with acute cholecystitis to help optimize management.

## Conclusions

CCI is a useful score that can be used as a preoperative predictor score for difficult cholecystectomy. It can help the surgeon decide on the optimum management plan for patients admitted with acute cholecystitis. Further studies are needed to determine how to better predict outcomes of this common surgical disease.
